# Novel Dioxygenases, HIF-α Specific Prolyl-hydroxylase and Asparanginyl-hydroxylase: O_2_ Switch for Cell Survival

**DOI:** 10.5487/TR.2008.24.2.101

**Published:** 2008-06-01

**Authors:** Hyunsung Park

**Affiliations:** Department of Life Science, University of Seoul, Tongdaemun-gu, Seoul, 130-743 Korea

**Keywords:** Hypoxia, HIF, PHD, FIH-1, Dioxygenase

## Abstract

Studies on hypoxia-signaling pathways have revealed novel Fe(II) and α-ketoglutarate-dependent dioxygenases that hydroxylate prolyl or asparaginyl residues of a transactivator, Hypoxia-Inducible Factor-α (HIF-α) protein. The recognition of these unprecedented dioxygenases has led to open a new paradigm that the hydroxylation mediates an instant post-translational modification of a protein in response to the changes in cellular concentrations of oxygen, reducing agents, or α-ketoglutarate. Activity of HIF-α is repressed by two hydroxylases. One is HIF-α specific prolyl-hydroxylases, referred as prolyl-hydroxylase domain (PHD). The other is HIF-α specific asparaginyl-hydroxylase, referred as factor-inhibiting HIF-1 (FIH-1). The facts (i) that many dioxygenases commonly use molecular oxygen and reducing agents during detoxification of xenobiotics, (ii) that detoxification reaction produces radicals and reactive oxygen species, and (iii) that activities of both PHD and FIH-1 are regulated by the changes in the balance between oxygen species and reducing agents, imply the possibility that the activity of HIF-α can be increased during detoxification process. The importance of HIF-α in cancer and ischemic diseases has been emphasized since its target genes mediate various hypoxic responses including angiogenesis, erythropoiesis, glycolysis, pH balance, metastasis, invasion and cell survival. Therefore, activators of PHDs and FIH-1 can be potential anticancer drugs which could reduce the activity of HIF, whereas inhibitors, for preventing ischemic diseases. This review highlights these novel dioxygenases, PHDs and FIH-1 as specific target against not only cancers but also ischemic diseases.

***Hypoxia-Inducible Factor α/β is a master transactivator for hypoxia-inducible genes.*** In response to lack of oxygen, cells induce several genes to maintain homeostasis. Hypoxia increases neovascularization, erythropoiesis, glycolysis, $H^+$ export, and vasodilation by inducing the corresponding genes including vascular endothelial growth factor (VEGF), erythropoietin (EPO), phosphoglycerate kinase-1 (PGK-1), glucose transporter-1 (GLUT-1), carbonic anhydrase 9 (CA9) and inducible NO synthase (iNOS) (Semenza, 2007). These diverse genes are transcribed by a common transactivator, named hypoxia-inducible factor-α/β (HIF-α/β) (Fig. 1). HIF-1 was first identified as a heterodimeric transactivator composed of two subunits, HIF-1α and β, both of which belong to the family of basic-helix-loop-helix-PAS

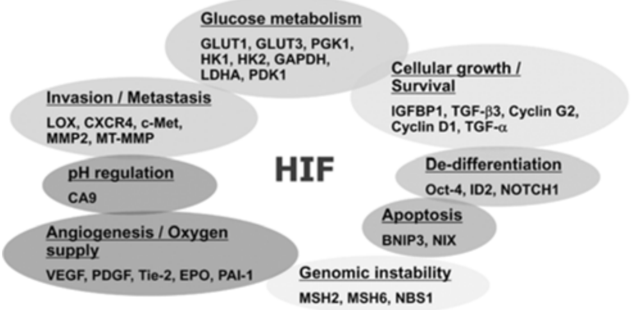

**Fig. 1.** Target genes of hypoxia-inducible factor

(bHLH-PAS) proteins, including Period (Per), aryl hydrocarbon receptor nuclear translocator (Arnt) and Single-minded (Sim) (Fig. 2). The bHLH-PAS proteins share common characteristics; first, a bHLH-PAS protein dimerizes with a specific partner protein through the HLH-PAS domain. Second, aryl hydrocarbon receptor (AhR)

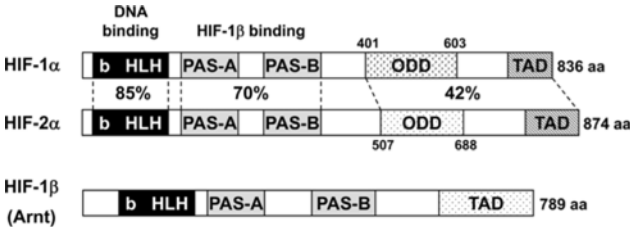

**Fig. 2.** Schematic structures of human HIF-1α, -2α and HIF-1β (Arnt). bHLH, basic helix-loop-helix domain; PAS, Per-Arnt-Sim domain; ODD, oxygen dependent degradation domain; TAD, transactivation domain.

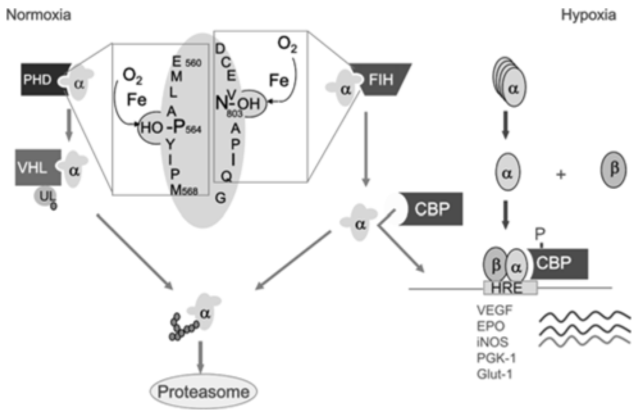

**Fig. 3.** Activation process of HIF-1α

or HIF-1α is activated by specific stimuli i.e., xenobiotics or low oxygen tension, respectively, before translocating to the nucleus where it heterodimerizes with a partner protein. Alternatively, Arnt, another bHLH-PAS protein, is constitutively located in the nucleus and interacts with several bHLH-PAS proteins in diverse pathways. Arnt, identical to HIF-1β, is known to be a common partner protein of AhR, mouse single-minded (mSim) and HIF-1α. Third, interactions between HLH-PAS domains bring two basic regions of the two subunits into juxtaposition, enabling individual basic regions to contact specific corresponding DNA sequences. Thus, dimerization of bHLH-PAS proteins is a prerequisite for DNA binding (Crews, 1998). HIF-1α was the original HIF-α isoform identified by affinity purification, while HIF-2α was identified in a homology search (Tian *et al.*, 1998). Both HIF-1α and HIF-2α form functional heterodimers with Arnt. HIF-1α protein is rapidly accumulated in acute hypoxia, and then declines after prolonged hypoxia, whereas HIF-2α protein increases slowly but continuously in prolonged hypoxia (Holmquist-Mengelbier *et al.*, 2006).

***Oxygen-dependent regulation of HIF-1α.*** Exposure to hypoxia for less than 2 min already revealed nuclear HIF-1α protein. One hour after hypoxic exposure, nuclear HIF-1α proteins reached maximal levels. HIF-1α induction is very instant in response to oxygen level (Jawell, *et al.*, 2001). In normoxia, HIF-1α is ubiquitinated and rapidly degraded while HIF-1β (Arnt) is constitutively expressed. The stability and activity of the α subunit of HIF-1 are regulated by posttranslational modification, specifically, by hydroxylation. Proline residues 402 and 564 of the oxygen-dependent degradation domain (ODD, amino acids 401-603 of human HIF-1α) are hydroxylated, mainly by HIF-1α-specific proly-4-hydroxylase (PHD) using molecular oxygen, α-ketoglutarate, vitamin C (Vit. C), and Fe(II) (Eptein *et al.*, 2001). The hydroxylated prolines are recognized by the E3 ubiquitin ligase, von Hippel-Lindau protein (pVHL), following which HIF-1α is polyubiquitinated and degraded by the 26S proteasomal system (Masson *et al.*, 2001). Hypoxia-induced *trans*-activation ability of HIF-1α is regulated by different mechanisms than its stabilization. To be a functional transactivator, the stabilized HIF-1α should be able to recruit its coactivator, CBP/p300. Asparagine 803 of human HIF-1α is also hydroxylated under normoxic conditions by an oxygen-dependent asparagine hydroxylase, referred to as Factor-Inhibiting HIF-1 (FIH-1). The hydroxylated asparagine residue hinders the recruitment of CBP/p300, thereby inhibiting transactivation by the stabilized HIF-1α (Fig. 3). A lack of oxygen reduces the activities of these two oxygen-dependent hydroxylases, so stabilizing the transactive form of HIF-1α (Hewitson *et al.*, 2002; Lando *et al.*, 2002). The findings of PHD and FIH-1 explain how HIF-1α is instantly activated with a few minutes in response to hypoxia. Therefore these dioxygenases are the ultimate $O_2$ sensors for hypoxic induction of genes.

***PHDs and FIH-1.*** By using a genetic approach in the roundworm *C. elegans*, Epstein *et al.* (2001) identified VHL and HIF-1α homologs. The investigators searched the *C. elegans* genome database for sequences that might encode a member of the α-ketoglutarate-dependent oxygenase superfamily and identified egl-9 gene, which stimulates the interaction between VHL and HIF-1α. Using the egl-9 sequences, three mammalian homologs of egl-9 were identified, and referred as PHD 1, 2 and 3. By using siRNA against PHD1, 2 or 3, PHD2 is found to be the major HIF-1α prolyl-4-hydroxylase (Berra *et al.*, 2003). PHD2 shares the conserved catalytic domain of α-ketoglutarate-dependent dioxygenases with other prolyl-4 hydroxylases including PHD1, PHD3 and collagen prolyl hydroxylase, but has a unique N-terminal

MYND-type zinc finger domain which inhibits the catalytic activity of PHD2 (Choi *et al.*, 2005).

FIH-1 has been identified as a protein that interacts with both HIF-1α and VHL, and that inhibits transactivation function of HIF-1α (Mahon *et al.*, 2001). Later, Lando *et al.* (2002) first found that FIH-1 is an asparaginyl hydroxylase. Asparagine-803 in the C-terminal transactivation domain of HIF-1α is hydroxylated by FIH-1 under normoxic conditions causing abrogation of the HIF-1α/p300 interaction (Hewitson *et al.*, 2002; McNeill *et al.*, 2002). Both PHDs and FIH-1 belong to the family of α-ketoglutarate-dependent dioxygenases, which also require $O_2$, Fe(II), and Vit. C for their reaction (Fig. 4).

The structure of FIH-1 is distinct from those of PHDs and collagen-prolyl-4-hydroxylase. The catalytic region of FIH-1 (145 to 340 amino acids of human FIH-1) belongs to the Jumonji (Jmj) domain which is often found in histone demethylases (Lee *et al.*, 2003; Klose *et al.*, 2006; Ng *et al.*, 2007). Jmj domains catalyze the oxidative demethylation of a methylated lysine residue of histone (Fig. 5). Although Jmj domain and FIH-1 hydroxylate different target proteins, both commonly utilize $O_2$, α-ketoglutarate, Vit. C and Fe(II) and produce succinate and $CO_2$. Both PHDs and FIH-1 hydroxylates HIF-α by using common cofactors, however FIH-1 has unique features in terms of structure and catalytic properties compared to PHDs (Hewitson *et al.*, 2007; Koivunen et al., 2007).

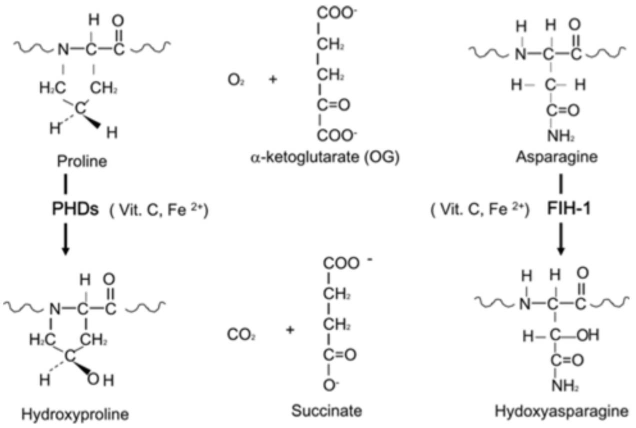

**Fig. 4.** Prolyl- and asparaginyl-hydroxylation by PHDs and FIH-1

***Km* values of PHDs and FIH-1 for their substrates.** *Km* values of PHDs and FIH-1 for their substrates were estimated by using purified recombinant hydroxylases which were expressed in insect cells (Table 1). The *Km* values of PHD1, 2 and 3 for $O_2$ are slightly above the $O_2$ concentration of the air (200 μM),

**Table 1.** *Km* values of PHDs, C4H, and FIH-1 for their substrates

| Enzyme | *Km* (μM) | | | Ref. |
|---|---|---|---|---|
| | α-Ketoglutarate | Ascorbate | $O_2$ | |
| PHD-1 | 60 | 170 | 230 | Hirsilä *et al.*, 2003 |
| PHD-2 | 60 | 180 | 250 | Hirsilä *et al.*, 2003 |
| PHD-3 | 55 | 140 | 230 | Hirsilä *et al.*, 2003 |
| FIH-1 | 25 | 260 | 90 | Koivunen *et al.*, 2004 |
| C4H1 | 20 | 300 | 40 | Vuorela *et al.*, 1997 |

[1]C4H, collagen prolyl-4-hydroxylase

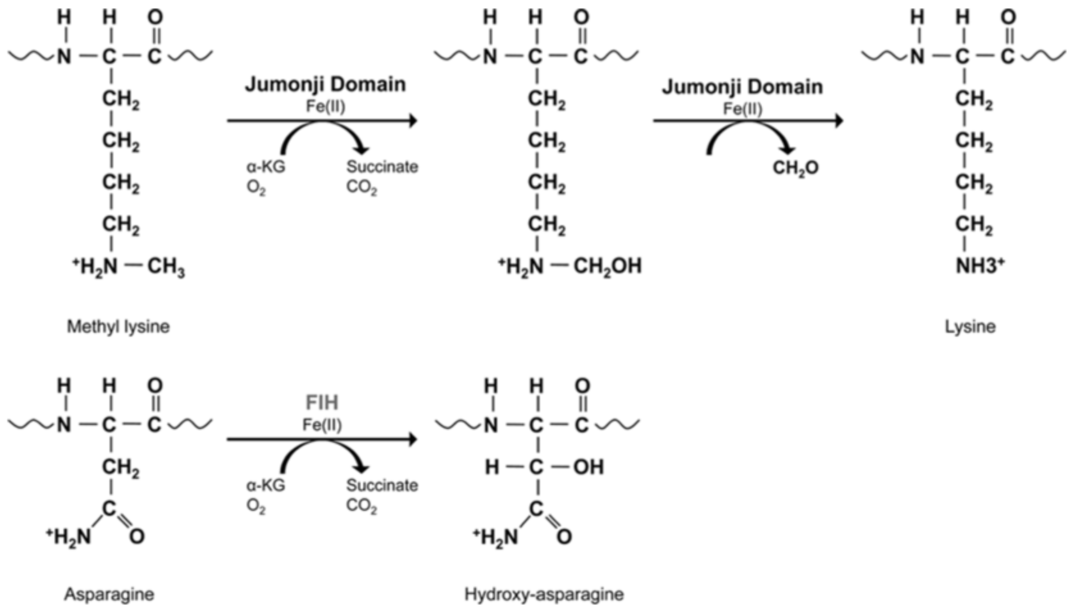

**Fig. 5.** Hydroxylation-dependent demethylation by Jumonji domain indicating that minor decease in oxygen effectively represses the activity of PHDs (Hirsilä *et al.*, 2003). In contrast, the *Km* values of FIH-1 and collagen prolyl-4 hydroxylase (C4H) for $O_2$ are 90 μM and 40 μM respectively, indicating that catalytic activities of FIH-1 and collagen prolyl-4 hydroxylase become half of maximum activity at 4.25% and 2% oxygen tension, respectively (Koivunen *et al.*, 2004; Vuorela *et al.*, 1997). Therefore, a larger decrease in $O_2$ concentration is required for a significant reduction in the activity of FIH-1, thus leading to full transactivation of HIF-1α (Koivunen *et al.*, 2007). Recent findings showed that differently from the cultured cells, $O_2$ concentration of mammalian tissue in a body is estimated about 2 to 10% oxygen tension, suggesting that the activities of both PHDs and FIH-1 are limited in a tissue (Brahimi-Horn and Pouysségur 2007). Therefore, in a normal tissue the basal level of HIF-1α may be higher than that of the cultured cells in 20% oxygen tension.

The fact that the catalytic activities of both PHDs and FIH-1 are related with the concentration of not only $O_2$ but also other co-substrates including α-ketoglutarate, Fe(II) and Vit. C, suggests that beside of hypoxia, any cellular events that cause the decrease in these co-substrates inhibits the activities of both PHDs and FIH-1. The *Km* values of PHDs for α-ketoglutarate are about two fold higher than those of FIH-1 and collagen prolyl-4 hydroxylase, indicating that they may show the distinct difference in their response to competitive inhibitors (Hewitson *et al.*, 2007; Hirsilä *et al.*, 2005; Nangaku *et al.*, 2006, Nytko *et al.*, 2007). Succinate and fumarate inhibit PHD but not FIH-1 activity (Hewitson *et al.*, 2007; Koivunen *et al.*, 2007). Desferrioxamine and several metals are effective inhibitors of FIH-1 but ineffective inhibitors of PHDs *in vitro* (Hirsilä *et al.*, 2005).

***Activators of PHD2 and FIH-1 as anticancer drugs.*** Tumors are frequently hypoxic due to the lack of blood supply and high oxygen consumption. Consequently, HIF is activated in most of cancers and contributes to survival and progression of cancers by triggering angiogenesis, glycolysis, $H^+$ export and metastasis (Esteban and Maxwell, 2005; Esteban *et al.*, 2006; Isaacs *et al.*, 2005; Koike *et al.*, 2004; Pouysségur *et al.*, 2006). Activators of PHDs and FIH-1 can be potential anticancer drugs which could reduce the activity of HIF (Choi *et al.*, 2008; Ginouvès *et al.*, 2008; Shin *et al.*, 2007, 2008). Inherited genetic defects in either fumarate hydratase (Selak *et al.*, 2005) or succinate dehydrogenase (Pollard *et al.*, 2005) cause tumor, however the mechanism of this cancer syndrome has not been known. The finding of PHD gave an answer. During dioxygenation reaction, PHDs catalyses HIF-α hydroxylation using α-ketoglutarate and generate succinate as a product. Loss-of-function mutations in fumarate hydratase or succinate dehydrogenase lead to the increase levels of succinate and fumarate. Then the excess succinate and fumarate could antagonize PHDs, leading to activation of HIF-α.

PHDs and FIH-1 also utilize Vit. C for their hydroxylation reaction. Vit. C and Vit. E are the major antioxidants which scavenge radicals such as reactive oxygen species. Anticancer activity of Vit. C has been presumed to arise from its ability to scavenge reactive oxygen species which cause DNA damage. Gao *et al.* (2007) showed that ectopic expression of stabilized HIF-1 mutant rescued the tumor from inhibition by Vit. C, indicating that Vit. C suppresses tumorigenesis primarily by activating PHDs rather than deceasing DNA damage.

***Inhibitors of PHDs and FIH-1, for ischemic diseases.*** HIF-1 attenuates ischemic injury. The beneficial effects of HIF-1 are mostly mediated by its target genes, the most prominent of which are EPO and VEGF, which are considered major mediators of the protective effect of hypoxic preconditioning (Baranova *et al.*, 2007; Grimm *et al.*, 2002; Natarajan *et al.*, 2006). Therefore, inhibitors of both PHDs and FIH-1 can be useful as an inducer of HIF-α and its target genes in ischemic diseases such as myocardial or cerebral infarction. Choi *et al.* (2006) found that the Cu(II) and Zn(II)-specific chelator Clioquinol (10~50 μM) increases functional HIF-1α protein, leading to increased expression of its target genes, VEGF and EPO. Interestingly, Clioquinol prevents FIH-1 from hydroxylating the asparagines residue (803), Cu(II)- and Zn(II)-independently. In contrast, Clioquinol fails to prevent PHD2 from hydroxylating proline. Instead, it blocks ubiquitination of the proline-hydroxylated HIF-1α in Cu(II)- and Zn(II)-dependent manner, thereby clioquinol stabilizes the *trans*-active form of HIF-1α (Choi *et al.*, 2006).

The mechanisms by which NO donors activate the function of HIF-1α are very diverse (Brüne and Zhou, 2007). NOC18 stimulates translation of HIF-1α by activating the PI3 kinase and Akt pathways (Kasuno *et al.*, 2004). Park *et al.* (2008) demonstrated that NO donor, SNAP inhibits FIH-1 but not PHD2. Instead, it inhibits VHL recruitment, through S-nitrosylation of ODD. Thus SNAP stabilizes the trans-active form of HIF-1α in normoxic cells. Interestingly, these inhibitory effects of SNAP can be reversed by addition of reducing agents such as excess Vit. C or Fe(II) indicating that cellular redox status affects SNAP action, presumably NO release from SNAP. Consistently, Li *et al.* (2007) demonstrated, using an antibody directed against the hydroxylated proline of HIF-1α, that in GSNO-treated mouse 4T tumor cells, proline-hydroxylated HIF-1α remained detectable. Biotin switch assay showed that a cysteine residue of ODD domain is nitrosylated, and that this S-nitrosylation does not inhibit proline hydroxylation but blocks VHL recruitment.

***Other substrates of PHDs and FIH-1.*** The findings of PHDs and FIH-1 have raised a question whether these hydroxylases have alternative substrates. Other substrates for PHD2 have not been found yet. However, Poellinger's group showed that FIH-1 hydroxylates Notch at two residues ($N^{1945}$ and $N^{2012}$) (Zheng *et al.*, 2008). In a screen for proteins interacting with FIH-1, Ratcliffe's group also has show that FIH-1 also efficiently hydroxylates specific asparaginyl (Asn)-residues of other ankyrin repeat domain-containing proteins such as NFκB and IκBα (Cockman *et al.*, 2006). These findings demonstrate that this type of protein hydroxylation is not restricted to HIF and strongly suggest that FIH-dependent ankyrin repeat domain (ARD) hydroxylation is a common occurrence, potentially providing an oxygen-sensitive signal to a diverse range of processes.

## ACKNOWLEDGEMENT

This study was supported by a research grant (2007) from the University of Seoul to H. Park.
